# Effects of Herbal and Natural Product Interventions on Gut Microbiota and Clinical Outcomes in Patients Receiving PPI-Containing Therapy: A Systematic Review and Meta-Analysis

**DOI:** 10.3390/nu18111792

**Published:** 2026-06-02

**Authors:** Ji Hye Hwang, You-Kyung Choi

**Affiliations:** 1 Department of Acupuncture & Moxibustion Medicine, College of Korean Medicine, Gachon University, Seongnam 13120, Republic of Korea; 2 Department of Korean Internal Medicine, College of Korean Medicine, Wonkwang University, Iksan 54538, Republic of Korea; 3 Kyunghee Hyojeon Korean Medicine Clinic, Seoul 03176, Republic of Korea

**Keywords:** herbal medicine, natural products, gut microbiota, proton pump inhibitors, dysbiosis, *Helicobacter pylori*, systematic review

## Abstract

Proton pump inhibitor (PPI)-containing regimens, including bismuth quadruple therapy, may perturb gut microbiota through combined exposure to acid suppression, antibiotics, bismuth, and underlying disease context. Herbal medicines and natural products have been proposed as adjunctive interventions to mitigate treatment-related microbiota perturbations; however, systematic synthesis of the clinical evidence remains limited. This systematic review and meta-analysis evaluated the effects of herbal and natural product interventions on gut microbiota and clinical outcomes in patients receiving PPI-containing therapy. Six databases (PubMed, EMBASE, Web of Science, Scopus, CENTRAL, and CNKI) were searched from their inception to March 2026. Risk of bias was assessed using RoB 2.0 and ROBINS-I. This review was prospectively registered in PROSPERO (CRD420261346672). Eighteen studies (17 randomized controlled trials, 1 observational study; *n* = 1984 participants) were included in the final analysis. Meta-analysis demonstrated significantly higher *Helicobacter pylori* eradication rates (pooled relative risk (RR) = 1.20, 95% confidence interval (CI) 1.14–1.27; *I*^2^ = 33%). Chinese-style total effective rate was also higher in the herbal groups (RR = 1.19, 95% CI 1.14–1.25; *I*^2^ = 0%), but this non-standardized outcome should be interpreted cautiously. Exploratory microbiome meta-analyses suggested higher post-treatment *Bifidobacterium* and *Lactobacillus* levels; however, substantial heterogeneity limited interpretability. Narrative synthesis revealed potential preservation of α-diversity and attenuation of pathobiont proliferation in herbal groups. Overall, herbal and natural product interventions may be associated with favorable clinical outcomes and potential microbiota-modulating effects in patients receiving PPI-containing therapy, but certainty remains limited due to methodological concerns, outcome indirectness, and heterogeneity. High-quality trials stratified by antibiotic exposure are warranted.

## 1. Introduction

Intestinal homeostasis is fundamentally governed by a complex microbial ecosystem, the disruption of which is termed dysbiosis and serves as the primary catalyst in diverse systemic pathophysiologies [1,2]. Proton pump inhibitors (PPIs) have emerged as a major concern among the pharmacological factors that influence this delicate balance. Although PPIs remain the cornerstone of therapy for acid-related disorders, their widespread use is increasingly associated with profound alterations in gastrointestinal ecology [3,4,5]. By elevating the gastric pH, PPIs compromise the innate acid barrier, facilitating the ectopic colonization of oral-derived taxa and environmental pathobionts within the lower gastrointestinal tract [5,6]. Translocation of oral microorganisms into the gut is clinically significant because oral microbial dysbiosis has been independently linked to systemic inflammatory, metabolic, and neurodegenerative disorders [7].

The clinical complexity of this issue is further exacerbated by the management of *Helicobacter pylori* (*H. pylori*) infections. In these scenarios, acid suppression is rarely an isolated intervention; rather, it is co-administered with broad-spectrum antibiotics in bismuth quadruple or triple-therapy regimens [8]. This combined pharmacological exposure triggers a synergistic disruption of the gut microbiota, characterized by a rapid decline in alpha diversity and an expansion of opportunistic pathogens [9,10]. Importantly, the resulting dysbiosis reflects the composite effect of both acid suppression and antibiotic-induced perturbation, a distinction that is clinically relevant when evaluating microbiota-targeted interventions in this context.

Despite the evident risks of treatment-induced dysbiosis, current therapeutic protocols predominantly focus on pathogen eradication and acid neutralization, often neglecting the need for ecological recovery [9]. Although probiotics have been used as adjunctive measures, their efficacy in achieving sustained microbial restoration following dual antibiotic–PPI exposure remains inconsistent [9,11]. This gap in care highlights the critical need for multifaceted interventions to promote a more resilient microbial environment.

Herbal medicines and natural products may represent potentially relevant and mechanistically distinct approaches. Characterized by their multicomponent profiles, these interventions may exert broader ecological effects on the gut microbiome through multiple complementary mechanisms, moving beyond simple microbial replacement [4,12]. Proposed mechanisms include prebiotic-like stimulation of butyrate-producing commensals, modulation of microbial metabolic pathways, and restoration of host–microbiome crosstalk [12,13]. Furthermore, the bidirectional metabolism of herbal constituents, wherein the microbiota transform parent compounds into bioactive metabolites, may contribute to symbiotic interactions between these interventions and the gut ecosystem [14,15]. Many of the interventions evaluated in this review were derived from traditional East Asian medical systems, which have long utilized multicomponent formulations for the management of gastrointestinal disorders. This ethnopharmacological background provides a rationale for investigating their potential role in modulating gut microbiota under conditions of pharmacological perturbation. However, existing clinical evidence for these effects remains fragmented across heterogeneous disease populations and assessment methodologies. Although a preliminary protocol for this review was prospectively registered and published [16], a comprehensive and integrated synthesis of clinical outcomes and microbiome structural shifts in the specific context of acid suppression-associated dysbiosis is currently lacking.

Therefore, this systematic review and meta-analysis were conducted to bridge this gap. We aimed to evaluate the effects of herbal medicines and natural products on gut microbial composition and clinical outcomes in patients receiving PPI-containing therapy. This study seeks to provide a rigorous evidence-based framework for integrating these interventions into standard gastrointestinal care, with the aim of mitigating the unintended microbiological consequences of pharmacological treatment.

## 2. Materials and Methods

This systematic review and meta-analysis were conducted in accordance with the Preferred Reporting Items for Systematic Reviews and Meta-Analyses (PRISMA 2020) guidelines [17] and the Synthesis Without Meta-Analysis (SWiM) reporting guidelines [18] for narrative outcomes. The review protocol was prospectively registered with the PROSPERO International Prospective Register of Systematic Reviews (registration number: CRD420261346672).

### 2.1. Identification of Eligible Studies

#### 2.1.1. Types of Study

Randomized controlled trials (RCTs) were eligible for inclusion. Non-randomized comparative studies were considered only if they provided quantitative microbiome data unavailable from RCTs. In such cases, the risk of bias was assessed using the ROBINS-I tool [19] rather than RoB 2.0 [20]. Single-arm pre-post studies were included only for narrative synthesis and were excluded from all quantitative pooling.

#### 2.1.2. Participants

Adult patients (aged ≥18 years) with any acid suppression-related gastrointestinal condition, including *H. pylori*-associated gastritis, gastric or duodenal ulcer, non-erosive reflux disease (NERD) [21], refractory gastroesophageal reflux disease (rGERD) [22], chronic atrophic gastritis (CAG), and erosive gastritis, were eligible. Studies were eligible regardless of whether participants were concurrently receiving PPI monotherapy or bismuth quadruple antibiotic therapy. Where antibiotic co-administration was present, the observed gut microbiota changes reflected the combined effects of acid suppression, antibiotic exposure, bismuth, and underlying disease context, and cannot be attributed solely to PPI-induced acid suppression [23].

#### 2.1.3. Interventions

Eligible interventions included any herbal medicine preparation (single-herb, multi-herb formulation, or standardized extract) or natural products (including marine-derived polysaccharides and botanical preparations) administered as adjuncts to, or in place of, standard pharmacological acid suppression therapy. Studies evaluating herbal medicines in combination with probiotic or synbiotic preparations were eligible if the herbal component was the primary study intervention.

#### 2.1.4. Comparators

The eligible comparators included (1) bismuth quadruple therapy alone (PPI plus two antibiotics plus bismuth), (2) PPI monotherapy, (3) PPI plus prokinetic therapy, and (4) no active comparator (single-arm designs, included for narrative synthesis only).

#### 2.1.5. Outcome Measures

Primary outcomes: *H. pylori* eradication rate (intention-to-treat analysis preferred); Chinese-style clinical total effective rate, defined as the proportion of patients achieving any of three response levels (clinical cure, marked improvement, or improvement) as evaluated according to standardized Chinese medicine outcome criteria [24]. Because this composite outcome is not directly equivalent to internationally standardized symptom-based endpoints, it was interpreted as a region-specific supportive clinical outcome rather than as a universally validated efficacy endpoint.

Secondary outcomes: incidence of adverse events and recurrence rate at follow-up.

Exploratory outcomes: Quantitative changes in *Bifidobacterium* and *Lactobacillus* abundance (lgCFU/g or logCFU/g); direction and magnitude of α-diversity and β-diversity changes; phylum-level and genus-level taxonomic shifts; metabolomic pathway changes.

### 2.2. Information Sources and Search Strategy

A systematic literature search was conducted in PubMed/MEDLINE, EMBASE, Web of Science, Scopus, Cochrane Central Register of Controlled Trials (CENTRAL), and China National Knowledge Infrastructure (CNKI) without language restrictions. CNKI was included because a substantial proportion of clinical trials evaluating herbal medicine interventions were conducted and published in East Asia and may not be indexed in Western databases [25]. The search covered records published between inception and March 2026. The search terms included MeSH headings and free-text terms for herbal medicines, traditional Chinese medicines, natural products, PPIs, *Helicobacter pylori*, gut microbiota, and dysbiosis. The full search strategy is presented in Appendix A, Table A1.

### 2.3. Study Selection

**Figure 1:**
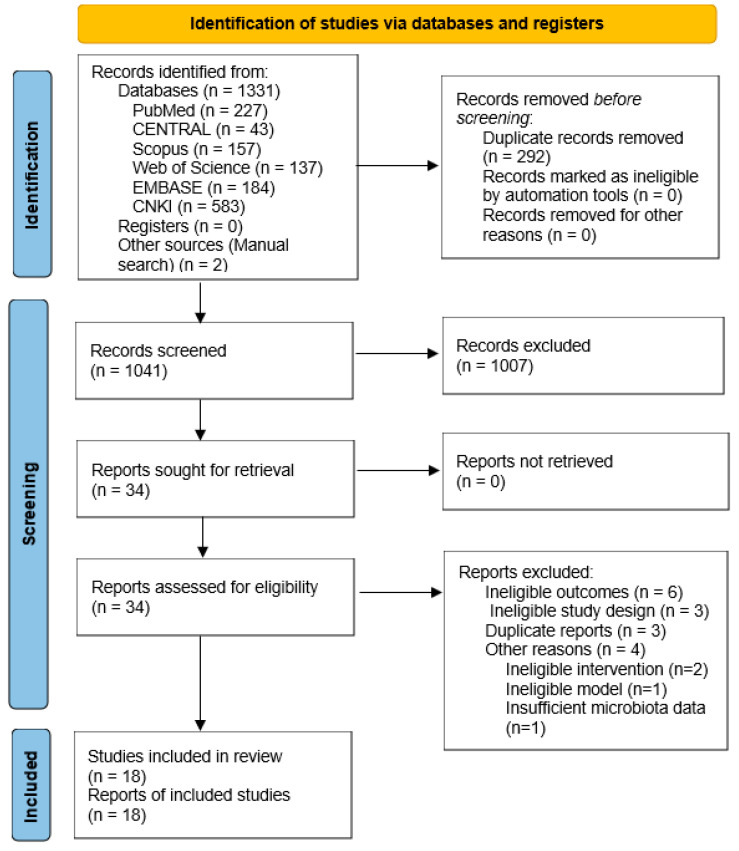
PRISMA 2020 flow diagram of the study selection process.

Two independent reviewers screened the titles and abstracts of the unique records identified through a systematic search, followed by a full-text assessment of potentially eligible reports. In accordance with Preferred Reporting Items for Systematic Reviews and Meta-Analyses (PRISMA) 2020, we distinguished between the number of unique studies and the number of corresponding reports during the selection process. Any disagreements were resolved by consensus or arbitration by a third reviewer. A PRISMA 2020 flow diagram documenting the screening process, including the reasons for exclusion at the full-text stage, is presented in Figure 1.

### 2.4. Data Extraction

Data were independently extracted by two reviewers using a pre-piloted standardized extraction form. The extracted variables included study design and randomization method, participant characteristics and disease population, intervention components, dose and duration, comparator regimen, antibiotic exposure status (yes/no), sample type for microbiome assessment (fecal vs. gastric), microbiome measurement methodology, outcome data with measures of precision, dietary variation or restrictions if reported, and risk of bias indicators. For microbiome data, the following fields were extracted separately: α-diversity indices (Shannon, Chao1, Simpson, Sobs), β-diversity analysis method and result, phylum-level and genus-level changes, and quantitative data for target taxa (*Bifidobacterium*, *Lactobacillus*) with pre- and post-treatment means and standard deviations per group.

### 2.5. Risk of Bias Assessment

The risk of bias in the RCTs was assessed using the Cochrane Risk of Bias 2.0 (RoB 2.0) tool, evaluating five domains: (D1) bias arising from the randomization process (including sequence generation and allocation concealment), (D2) bias because of deviations from intended interventions, (D3) bias because of missing outcome data, (D4) bias in the outcome measurement, and (D5) bias in the reported result selection. Each domain was rated as low risk, some concerns, or high risk, and an overall judgment was derived.

Non-randomized studies were assessed using the Risk of Bias in Non-randomized Studies of Interventions (ROBINS-I) tool and included for narrative synthesis only; they were excluded from all quantitative pooling.

### 2.6. Data Synthesis and Statistical Analysis

#### 2.6.1. Quantitative Meta-Analysis

Where sufficient data were available, a random-effects meta-analysis was performed using the DerSimonian–Laird method [26], accounting for between-study heterogeneity. For dichotomous outcomes (*H. pylori* eradication rate and total effective rate), pooled risk ratios (RR) with 95% confidence intervals (CI) were calculated using the Mantel–Haenszel method. For continuous outcomes (*Bifidobacterium* and *Lactobacillus* abundance), the standardized mean difference (SMD) with a 95% CI was used, given the substantial methodological heterogeneity in the measurement platforms across the studies.

Microbiome outcomes were pooled only when between-group comparisons were reported, and numerical data were sufficient for effect size estimation. Studies reporting non-significant between-group differences or insufficient quantitative data were excluded from quantitative synthesis and retained for narrative interpretation.

For multi-arm trials, only one pairwise comparison was included to avoid double-counting of participants. A comparison with the most clinically relevant intervention (e.g., quadruple therapy vs. combination therapy) was selected a priori.

#### 2.6.2. Outcome Classification and Pooling Eligibility

Western-style outcomes (e.g., symptom score reduction and visual analog scale (VAS)-based non-inferiority response) were not pooled with the Chinese-style total effective rate owing to conceptual and methodological heterogeneity in outcome definition and measurement.

Studies employing non-Chinese-style outcome definitions (e.g., the Leeds Dyspepsia Questionnaire, GERD-Q-based response, or VAS-based non-inferiority response) were excluded from the total effective rate meta-analysis and contributed to the narrative synthesis and NERD/GERD-specific subgroup analyses.

#### 2.6.3. Heterogeneity and Subgroup Analyses

Statistical heterogeneity was assessed using the Cochran Q test and *I*^2^ statistic, with *I*^2^ > 50% indicating substantial heterogeneity. Where heterogeneity was substantial, the sources were explored through pre-specified subgroup analyses.

Owing to the methodological heterogeneity in microbiome assessment platforms (culture-based enumeration, reverse transcription-polymerase chain reaction [RT-PCR]/reverse transcription quantitative real-time polymerase chain reaction [RT-qPCR], and automated mass spectrometry-based identification [Bruker matrix-assisted laser desorption/ionization time-of-flight mass spectrometry {MALDI-TOF}]), subgroup and sensitivity analyses were stratified by the measurement method. The SMD metric was selected to account for differences in scale and baseline value ranges across the measurement platforms.

Pre-specified subgroup analyses were stratified by (1) disease population (*H. pylori*-associated gastritis vs. gastric ulcer vs. erosive gastritis), (2) intervention type (multi-herb formulation vs. single natural compound vs. herbal plus probiotic/synbiotic combination), (3) antibiotic exposure status (quadruple therapy vs. PPI monotherapy), and (4) overall risk of bias (low vs. some concerns). Given the limited number of studies in each subgroup and the non-overlapping disease populations and outcome structures between PPI monotherapy and bismuth quadruple therapy contexts, formal subgroup meta-analyses by antibiotic exposure status were not performed. Instead, findings were stratified narratively according to antibiotic exposure where relevant.

#### 2.6.4. Narrative Synthesis

Outcomes for which quantitative pooling was not feasible were synthesized narratively, guided by the synthesis without meta-analysis (SWiM) framework [18]. These included: α-diversity indices (Shannon, Chao1, Sobs, Simpson); β-diversity and community structure; *Firmicutes*/*Bacteroidetes* ratio; phylum-level and genus-level taxonomic shifts; metabolomic pathway changes; and microbiome data from studies reporting gastric rather than fecal microbiota, which were analyzed as separate narrative subgroups and not pooled with fecal quantitative data.

Quantitative pooling of α-diversity indices was not performed. Numerical values were insufficiently reported across studies, measurement indices were heterogeneous (Shannon, Chao1, Sobs, and Simpson used variably), and assessment time points differed substantially between studies. α-Diversity data are therefore synthesized narratively, with direction of change and statistical significance reported per study where available.

### 2.7. Sensitivity Analyses

The following pre-specified sensitivity analyses were planned to assess the robustness of primary meta-analytic estimates in accordance with recommendations from the Cochrane Handbook for Systematic Reviews of Interventions [27]:

(1) Exclusion of studies rated as having some concerns or a high overall risk of bias, retaining only low-risk studies to assess the influence of methodological quality on pooled estimates.
(2) Inclusion of multi-arm trials using all available pairwise comparisons with appropriate participant splitting to avoid unit-of-analysis errors.
(3) Studies reporting non-significant between-group differences to evaluate the impact of borderline or null findings on pooled estimates.

### 2.8. Publication Bias

Where 10 or more studies contributed to a single pooled outcome, publication bias was assessed through visual inspection of the funnel plot. Formal statistical testing (Egger’s test [28]) was not performed because of the limited number of eligible studies and the exploratory nature of the analyses. For outcomes with fewer than ten studies, formal assessment of publication bias was not performed, in accordance with the Cochrane Handbook recommendations [27].

### 2.9. Anticipated Methodological Limitations

Most of the included studies were single-center trials conducted in China, which may limit the generalizability of the findings to non-Asian populations and healthcare settings where herbal formulations are not routinely available.

The substantial heterogeneity in treatment duration (ranging from 14 days to 12 weeks) across the included studies may have contributed to the variability in microbiome outcomes and limited direct comparisons between studies.

Additional anticipated limitations include the predominance of open-label study designs (14 of 18 studies), which introduce the risk of performance and detection bias, particularly for subjectively assessed outcomes; the use of culture-based enumeration methods in a subset of studies, which provide targeted quantification of selected taxa but lack the community-level resolution of 16S rRNA sequencing; and the short follow-up duration in most studies (2–4 weeks), which precludes assessment of the durability of microbiome effects following treatment cessation. Future trials in this area would benefit from adherence to established microbiome reporting standards such as the Strengthening The Organization and Reporting of Microbiome Studies (STORMS) checklist [29].

### 2.10. Software

Meta-analyses were performed using Review Manager (RevMan) version 5.4.1 (Cochrane Collaboration, Copenhagen, Denmark, 2020). Risk of bias assessments (https://www.riskofbias.info/) were documented using the RoB 2.0 and ROBINS-I assessment tools. The narrative synthesis followed the SWiM reporting framework.

## 3. Results

### 3.1. Study Selection

A systematic literature search identified 1333 records from six databases (PubMed, n = 227; EMBASE, n = 184; Web of Science, n = 137; Scopus, n = 157; CNKI, n = 583; CENTRAL, n = 43; total n = 1331) and through manual search and citation tracking (n = 2). Following the removal of 292 duplicate records, 1041 unique records were screened based on title and abstract. Of these, 1007 records were excluded because they did not meet the predefined eligibility criteria (e.g., ineligible disease population, animal models, or non-interventional study design). A total of 34 reports [30,31,32,33,34,35,36,37,38,39,40,41,42,43,44,45,46,47] were retrieved and assessed for eligibility through full-text review. During this stage, 16 reports were excluded for the following reasons: ineligible outcomes (n = 6; no gut microbiota analysis or related microbial metrics reported); ineligible study design (n = 3; non-interventional studies including observational designs and reviews); duplicate reports (n = 3; secondary publications or overlapping datasets from the same trial); ineligible intervention (n = 2; probiotics or synbiotics alone without a primary herbal or natural product component); ineligible model (n = 1; In Vivoin vivo animal study); and insufficient microbiota data (n = 1; lacked quantitative measures such as mean and standard deviation required for pooling). Ultimately, 18 studies described in 18 reports met all inclusion criteria and were included in the systematic review and meta-analysis. The detailed screening process is illustrated in the PRISMA 2020 flow diagram (Figure 1).

### 3.2. Study Characteristics

Eighteen studies, described in 18 reports, were included in the final analysis (Table 1). These studies, published between 2020 and 2026, comprised 17 RCTs and one pre-post single-arm observational study (assessed using ROBINS-I). All the studies were conducted in China, except those conducted in Australia [30] and one multicenter study conducted in Italy [34] (13 hospitals). The total number of participants across all included studies was 1984, ranging from 43 in the single-arm study [30] to 275 in the multicenter trial [34].

Disease populations included: *H. pylori*-associated chronic gastritis (seven studies) [32,33,37,41,42,44,46], *H. pylori* infection (three studies) [35,38,39], NERD or refractory GERD (three studies) [31,36,43], gastric ulcer (one study) [40], erosive gastritis (one study) [47], CAG with intestinal metaplasia (one study) [45], and mixed/functional gastrointestinal conditions (two studies) [30,34]. The treatment duration ranged from 14 days to 12 weeks. Most studies (15 of 18) employed an open-label design, whereas three trials utilized a double-blind, double-dummy methodology and were rated as having a low overall risk of bias.

Herbal interventions were classified into three subgroups: multi-herb formulations (13 studies) [31,32,36,37,39,40,41,42,43,44,45,46,47], a single natural compound (one study of fucoidan) [35], and herbal/natural product plus probiotic or synbiotic combinations (two studies) [33,38]. Two investigations were analyzed separately: a pre-post, single-arm study involving a multicomponent Western herbal preparation [30] and a trial evaluating a natural polysaccharide preparation using a comparator design [34].

Microbiological assessment methods vary substantially between studies. Seven studies employed high-resolution 16S rRNA/rDNA sequencing [31,34,35,36,37,38,46], five used RT-PCR or RT-qPCR targeting specific taxa [39,41,43,44,47], four used culture-based enumeration [32,33,40,42], and one used automated MALDI-TOF mass spectrometry [45]. All studies assessed fecal microbiota, except for one [42] that evaluated gastric microbiota (percentage composition from gastric fluid and biopsy specimens) and was therefore analyzed in a separate narrative subgroup.

### 3.3. Risk of Bias and Certainty of Evidence

**Figure 2:**
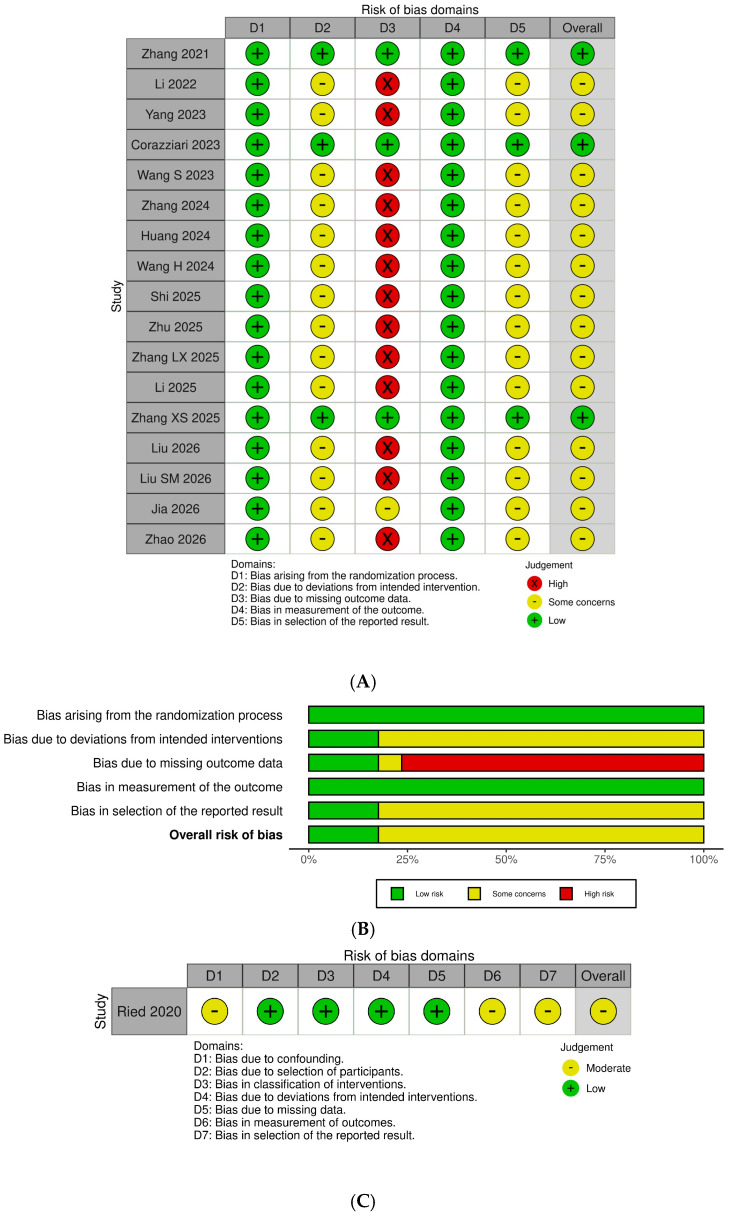
Risk of bias assessment of included studies. (**A**) Study-level risk of bias for randomized controlled trials (RCTs), assessed using the Cochrane Risk of Bias 2 (RoB 2) tool across five domains: bias arising from the randomization process (D1), bias because of deviations from intended interventions (D2), bias because of missing outcome data (D3), bias in measurement of the outcome (D4), and bias in selection of the reported result (D5). (**B**) Summary of risk of bias judgments across all included RCTs, presented as the proportion of studies rated as low risk, some concerns, or high risk for each domain. (**C**) Risk of bias assessment of the included non-randomized study (Ried et al., 2020) [30], evaluated using the ROBINS-I tool across seven domains, including confounding, participant selection, intervention classification, deviations from intended interventions, missing data, outcome measurement, and selective reporting. Zhang et al., 2021 [31]; Li et al., 2022 [32]; Yang et al., 2023 [33]; Corazziari et al., 2023 [34]; Wang et al., 2023 [35]; Zhang et al., 2024 [36]; Huang, 2024 [37]; Wang et al., 2024 [38]; Shi et al., 2025 [39]; Zhu and Tang, 2025 [40]; Zhang et al., 2025 [41]; Li et al., 2025 [42]; Zhang et al., 2025 [43]; Liu et al., 2026 [44]; Liu et al., 2026 [45]; Jia et al., 2026 [46]; Zhao and Sheng, 2026 [47].

Risk of bias assessment was performed using RoB 2.0 for 17 RCTs and ROBINS-I for the non-randomized study [30] (Figure 2). Three RCTs were judged to have a low overall risk of bias [31,34,43], all of which employed double-blind, double-dummy designs. The remaining 14 RCTs were rated as having some concerns. The primary source of concern was bias owing to deviations from intended interventions (D2), largely attributable to the open-label design and lack of blinding, which affected all 14 open-label trials. Bias arising from the randomization process (D1) was generally rated as low risk across the included RCTs, although the reporting of allocation concealment was often limited. Bias owing to missing outcome data (D3) was generally rated as low or concerning, although the reporting of attrition and data-handling procedures was often insufficient. Bias in the measurement of the outcome (D4) was generally rated as low risk, as most studies employed objective microbiome assessment methods, such as culture-based enumeration, PCR, and 16S rRNA sequencing. Bias in the selection of reported results (D5) was rated as a concern in 14 RCTs, reflecting the absence of pre-registered statistical analysis plans in most open-label studies. A single non-randomized study [30] was assessed as having a moderate overall risk of bias using ROBINS-I, primarily because of potential confounding factors and the absence of a comparator group.

**Figure 3:**
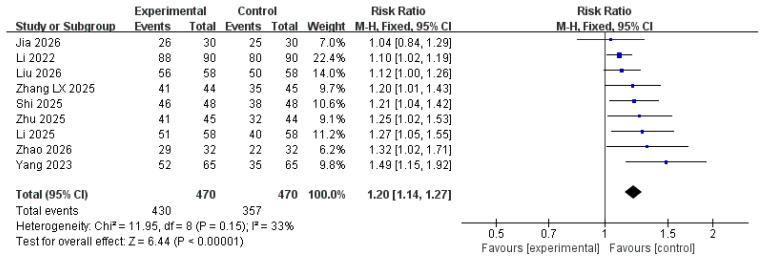
Forest plot of *H. pylori* eradication rates. Meta-analysis of nine studies comparing herbal intervention plus bismuth quadruple therapy versus bismuth quadruple therapy alone. Effect size expressed as risk ratio (RR) with 95% confidence intervals (CI) using a random-effects model (Mantel–Haenszel (M-H) method). Squares represent individual study effect estimates, with square size proportional to study weight. The diamond represents the pooled effect estimate and its 95% confidence interval. Li et al., 2022 [32], Yang et al., 2023 [33], Shi et al., 2025 [39], Zhu and Tang, 2025 [40], Zhang et al., 2025 [41], Li et al., 2025 [42], Liu et al., 2026 [44], Jia et al., 2026 [46], Zhao and Sheng, 2026 [47].

**Figure 4:**
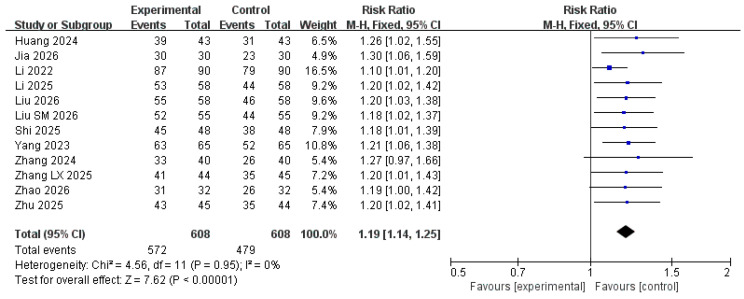
Forest plot of Chinese-style clinical total effective rates. Meta-analysis of 12 studies. Effect size expressed as risk ratio (RR) with 95% confidence intervals (CI) using a random-effects model (Mantel–Haenszel (M-H) method). Squares represent individual study effect estimates, with square size proportional to study weight. The diamond represents the pooled effect estimate and its 95% confidence interval. Li et al., 2022 [32], Yang et al., 2023 [33], Zhang et al., 2024 [36], Huang, 2024 [37], Shi et al., 2025 [39], Zhu and Tang, 2025 [40], Zhang et al., 2025 [41], Li et al., 2025 [42], Liu et al., 2026 [44], Liu et al., 2026 [45], Jia et al., 2026 [46], and Zhao and Sheng, 2026 [47].

**Figure 5:**
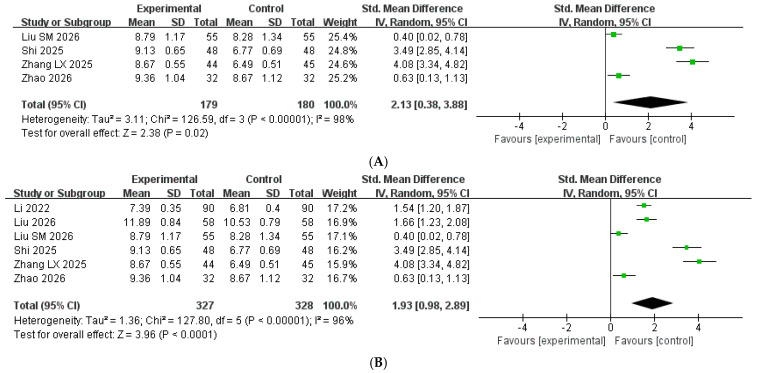
Forest plot of post-treatment *Bifidobacterium* abundance. (**A**) Main analysis (four studies: Shi et al., 2025 [39]; Zhang et al., 2025 [41]; Liu et al., 2026 [44]; Zhao and Sheng, 2026 [47]); (**B**) sensitivity analysis (six studies, additionally incorporating Li et al., 2022 [32] and Liu et al., 2026 [45]). Effect size expressed as standardized mean difference with 95% confidence intervals (CI) using a random-effects model (DerSimonian–Laird method). IV, inverse variance; SD, standard deviation, Squares represent individual study effect estimates, with square size proportional to study weight. The diamond represents the pooled effect estimate and its 95% confidence interval.

**Figure 6:**
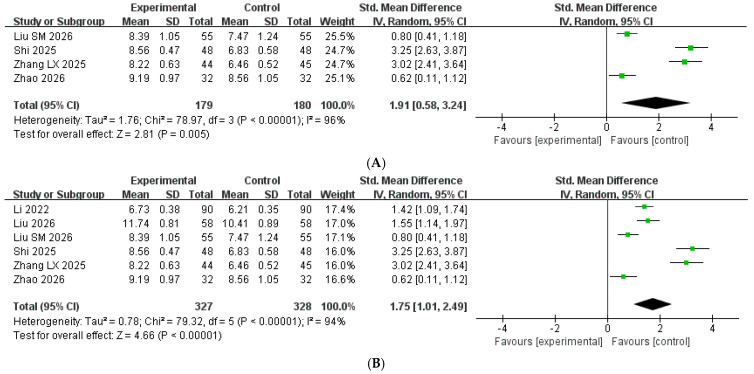
Forest plot of post-treatment *Lactobacillus* abundance. (**A**) Main analysis (four studies: Shi et al., 2025 [39]; Zhang et al., 2025 [41]; Liu et al., 2026 [44]; Zhao and Sheng, 2026 [47]); (**B**) sensitivity analysis (six studies, additionally incorporating Li et al., 2022 [32] and Liu et al., 2026 [45]). Effect size expressed as standardized mean difference with 95% confidence intervals (CI) using a random-effects model (DerSimonian–Laird method). IV, inverse variance; SD, standard deviation, Squares represent individual study effect estimates, with square size proportional to study weight. The diamond represents the pooled effect estimate and its 95% confidence interval.

Study labels in Figure 2, Figure 3, Figure 4, Figure 5 and Figure 6 are abbreviated as first author surname and year for visual clarity.

The certainty of evidence for the main pooled outcomes was assessed using the GRADE approach and is summarized in Table 2. The evidence for the *H. pylori* eradication rate was rated as low, primarily due to risk of bias from open-label study designs and indirectness. The evidence for Chinese-style total effective rate, *Bifidobacterium* abundance, and *Lactobacillus* abundance was rated as very low, reflecting additional concerns regarding the non-standardized nature of the total effective rate outcome and substantial statistical heterogeneity in microbiome outcomes (*I*^2^ = 96–98%).

### 3.4. H. pylori Eradication Rate

Nine studies [32,33,39,40,41,42,44,46,47] reported extractable *H. pylori* eradication data for between-group comparisons (Table 3). Eight of the nine studies reported a statistically significant superiority of the herbal intervention group over the bismuth quadruple therapy alone; the exception was one investigation [46] that showed a numerically higher but non-significant difference (86.67% vs. 83.33%; *p* > 0.05). Meta-analysis of these nine studies demonstrated a significantly higher *H. pylori* eradication rate in the herbal intervention group than in the control (pooled RR = 1.20, 95% CI 1.14–1.27; *I*^2^ = 33%; *p* < 0.00001; Figure 3), with low-to-moderate heterogeneity observed across studies.

Recurrence data were reported in two studies. One study [32] reported a 6-month recurrence rate of 1.15% (1/87) versus 10.39% (8/77) in favor of the herbal adjunct group (*p* = 0.024). The other investigation [46] also reported a lower recurrence rate in the herbal group (16.67% vs. 47.83%; *p* < 0.05), although the between-group *H. pylori* eradication rate difference was not statistically significant in that specific trial. These findings have been reported descriptively, given the limited number of studies assessing long-term outcomes.

### 3.5. Clinical Total Effective Rate

Twelve studies reported Chinese-style clinical total effective rates (clinical cure, marked improvement, or improvement) eligible for the primary total effective rate meta-analysis [32,33,36,37,39,40,41,42,44,45,46,47]. Four studies were excluded from this outcome pool because of conceptual and methodological heterogeneity: a pre-post study using the Leeds Dyspepsia Questionnaire [30], a trial focusing on the complete resolution rate [31], an investigation evaluating a VAS-based non-inferiority response [34], and a study utilizing a GERD-Q-based response [43]. The results of the 12 eligible studies are summarized in Table 4.

All 12 studies reported statistically significant differences favoring the herbal intervention group over the control group. The magnitudes of the benefits ranged from approximately 12–23%. Notably, two investigations [36,37] targeting those with rGERD and chronic gastritis, respectively, reported smaller absolute differences (17.5% and 18.6%) than studies conducted in *H. pylori* eradication contexts, reflecting potential disease-related heterogeneity. Meta-analysis of these 12 studies demonstrated a significantly higher total effective rate in the herbal intervention group than control (pooled RR = 1.19, 95% CI 1.14–1.25; *I*^2^ = 0%; *p* < 0.00001; Figure 4). No substantial heterogeneity was detected across the studies (*I*^2^ = 0%). However, because this outcome was derived from Chinese-style composite response criteria and most contributing trials were open-label, the apparent absence of heterogeneity should not be interpreted as definitive evidence of clinically uniform treatment effects.

### 3.6. NERD/GERD-Specific Outcomes

Three studies specifically targeted patients with NERD or rGERD [31,34,43]. One investigation [31] (evaluating JianpiQinghua granules plus low-dose omeprazole) achieved a complete resolution rate of 40.8% compared with 26.8% with standard-dose omeprazole (OR = 1.88, 95% CI: 1.03–3.44; *p* = 0.039). Another trial [43] demonstrated a total response rate of 90.00% versus 86.67% for omeprazole (*p* = 1.000, confirmed non-inferiority). The group receiving the herbal formula additionally showed statistically superior improvement in quality of life (Short Form Survey-36 Z = −3.271, *p* = 0.001) and cold-heat complex traditional Chinese medicine (TCM) syndrome scores, despite equivalent symptom response rates. A multicenter study [34] demonstrated the non-inferiority of the natural preparation for symptom relief assessed using VAS (difference −5.4 mm, 95% CI −9.9 to −0.1; ITT analysis); rescue medication use was significantly lower in the natural preparation group during weeks 2–4 (*p* = 0.019).

### 3.7. Gut Microbiota Outcomes

#### 3.7.1. α-Diversity

Seven studies assessed α-diversity using high-resolution sequencing [31,34,35,36,37,38,46]. Pooling of α-diversity data was not performed because of insufficient numerical reporting, heterogeneity of indices used, and differences in assessment time points. Results are therefore summarized narratively.

One study focusing on rGERD [36] reported statistically significant increases in Chao1, ACE, and Sobs indices after 8 weeks of herbal treatment compared with the control (*p* < 0.05). Another investigation [38] demonstrated that the bismuth quadruple therapy-alone group experienced significant decreases in Shannon and Chao1 indices at 4 weeks post-treatment (both *p* < 0.05), whereas neither the adjunctive supplementation group nor the synbiotics-only group exhibited significant diversity changes, indicating a buffering effect. A further trial [31] reported higher α-diversity in the combined herbal-PPI group than in PPI monotherapy, consistent with metabolomic evidence of corrected glutamate metabolism pathways. One investigation [46] reported directional increases in the Sobs, Chao1, Shannon, and Simpson indices in the herbal group, although the numerical values were not provided. Another study [43] reported that rarefaction curves reached saturation in both groups, suggesting adequate sequencing depth, although the numerical indices were not fully reported.

#### 3.7.2. β-Diversity and Community Structure

One study provided informative diversity data [43]. Principal coordinate analysis demonstrated that the gut microbiome configuration of patients with NERD was significantly different from that of healthy controls at baseline. After eight weeks of herbal treatment, the configuration converged toward the healthy control profile, whereas the omeprazole group retained a configuration significantly divergent from healthy controls. Another study [34] reported no significant within-group or between-group changes in α- or β-diversity in the natural preparation group (all *p* > 0.05), whereas the omeprazole group showed significantly increased Bray–Curtis dissimilarity from baseline to week 4, with specific enrichment of oral-derived genera (*Streptococcus salivarius*, *Streptococcus sinensis*, *Haemophilus parainfluenzae*, *Veillonella dispar*; all *p* < 0.0001).

#### 3.7.3. Bifidobacterium and Lactobacillus—Exploratory Analysis

Quantitative data on *Bifidobacterium* abundance were obtained from six studies (Table 5). Studies were eligible for exploratory pooling only when between-group comparisons were statistically significant and numerical data were sufficient for effect size estimation. Four studies met the criteria for exploratory pooling, and two additional studies contributed to sensitivity analyses. Two studies [32,44] were excluded from pooling: one [40] because of non-significant between-group comparisons, and another [33] because it reported attenuated dysbiosis rather than absolute increases.

In the four main exploratory studies, the herbal intervention group generally showed higher post-treatment *Bifidobacterium* levels than the control group. Notably, the control groups in two trials [39,41] showed actual decreases in *Bifidobacterium* following quadruple therapy, whereas herbal adjunct therapy not only prevented this decline but also produced significant increases. Exploratory meta-analysis of these four investigations suggested higher post-treatment *Bifidobacterium* abundance in the herbal intervention group (pooled SMD = 1.39, 95% CI 1.14–1.65; *I*^2^ = 98%; *p* < 0.00001; Figure 5A). However, substantial heterogeneity (*I*^2^ = 98%) considerably limited interpretability, and these findings should be regarded as exploratory.

A similar pattern was observed for *Lactobacillus* (Table 6). Exploratory meta-analysis of four investigations suggested higher post-treatment *Lactobacillus* abundance in the herbal intervention group (pooled SMD = 1.91, 95% CI 0.58–3.24; *I*^2^ = 96%; *p* = 0.005; Figure 6A). Sensitivity analysis incorporating two additional studies [32,44] yielded directionally similar results (pooled SMD = 1.75, 95% CI 1.01–2.49; *I*^2^ = 94%; *p* < 0.00001; Figure 6B). However, substantial heterogeneity indicates that these findings should be interpreted cautiously and considered hypothesis-generating rather than confirmatory.

#### 3.7.4. Gastric Microbiota

One study [42] (evaluating an herbal decoction plus bismuth quadruple therapy, n = 116) assessed gastric microbiota (percentage composition) rather than fecal gut microbiota, and was therefore analyzed separately. The herbal adjunct group showed a significantly greater increase in gastric *Lactobacillus* proportion (5.64% to 13.28%) than in the control group (6.05% to 11.57%; *p* < 0.001). Gastric *Streptococcus* proportion was significantly lower in the herbal medicine group (3.26%) than in the control group (4.21%; *p* < 0.001). Gastric *H. pylori* proportion decreased from 40.26% to 7.68% in the herbal medicine group and from 39.51% to 11.25% in the control group (*p* < 0.001). As these outcomes reflect gastric rather than fecal microbiota and are expressed as percentage proportions rather than absolute abundances (log CFU/g), they were not pooled with the fecal microbiome data from other studies.

#### 3.7.5. Pathobiont Suppression and Opportunistic Pathogen Changes

Across studies using quadruple antibiotic therapy as the comparator, control groups consistently showed increases in potentially pathogenic genera, including *Escherichia-Shigella*, *Enterococcus*, and *Staphylococcus* [33,38,39,44,45,47]. One investigation [38] demonstrated that only the combined fucoidan plus synbiotics group achieved complete suppression of *Escherichia-Shigella* proliferation and a significant reduction in *Klebsiella* (*p* = 0.009), effects not observed in the fucoidan-alone or synbiotics-alone groups. A further trial [34] confirmed that the PPI group showed significant enrichment of oral-derived genera (*Streptococcus salivarius*, *S. sinensis*, *Haemophilus parainfluenzae*, *Veillonella dispar*; all *p* < 0.0001), whereas the natural preparation produced no detectable microbiome perturbation.

### 3.8. Metabolomic Findings

Two studies incorporated metabolomic analyses alongside microbiome sequencing [31,43]. One study [31] identified a correction of the glutamate metabolism pathway in the herbal intervention group, an effect that was not observed with omeprazole monotherapy. Another investigation [43] demonstrated that the herbal formula reversed three disease-associated metabolic pathways in patients with NERD (tryptophan metabolism, glycine/serine/threonine metabolism, and neuroactive ligand-receptor interaction). Three differentially expressed metabolites, l-2,4-diaminobutyric acid, felbamate, and orphenadrine, were normalized toward healthy control levels following herbal treatment but not omeprazole, providing mechanistic evidence for the superior quality-of-life outcomes observed in the herbal intervention group.

### 3.9. Adverse Events

Adverse events were reported in 13 of 18 studies. The definitions and reporting practices for adverse events varied across studies, limiting direct comparisons. In most studies, the herbal intervention groups demonstrated lower adverse event rates than the control groups. Selected results included: 5.56% (5/90) vs. 14.44% (13/90) [32]; 2.33% (1/43) vs. 18.60% (8/43) [37]; 4.17% (2/48) vs. 16.67% (8/48) [39]; 7.27% (4/55) vs. 23.64% (13/55) (χ^2^ = 5.636, *p* = 0.018) [44]; and 6.8% (3/44) vs. 15.6% (7/45) [41]. Two studies reported comparable or higher adverse event rates in the herbal intervention group: one [33] (7.69% vs. 4.62%) and the other [40] (11.11% vs. 6.82%), both without a statistical comparison.

One study [31] reported an important safety signal that documented liver enzyme abnormalities in three participants in the combined herbal-PPI group (one mild and two moderate-to-severe). A multicenter trial [34] reported no serious adverse events in either group (natural preparations, 37.4% vs. PPI, 38.1%, all mild-to-moderate).

### 3.10. Publication Bias

**Figure 7:**
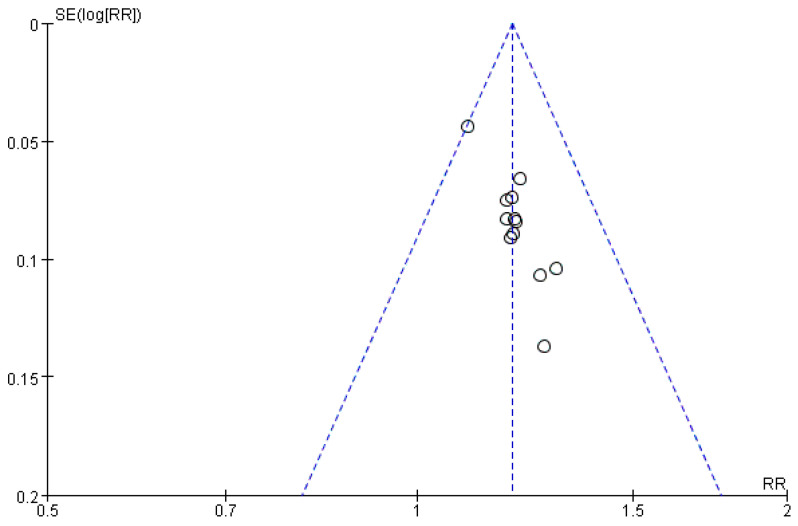
Funnel plot for total effective rate meta-analysis (12 studies). Visual inspection shows approximate symmetry, suggesting no major publication bias. RR, relative risk; SE, standard error. Open circles represent individual studies. The vertical dashed line indicates the pooled effect estimate, and the diagonal dashed lines represent the pseudo 95% confidence limits.

Potential publication bias for the primary outcome (total effective rate) was assessed through visual inspection of funnel plots (Figure 7). The funnel plot involving the 12 studies displayed an approximately symmetrical distribution, with studies evenly distributed on both sides of the pooled effect size and clustered toward the top of the plot. This visual symmetry, combined with the lack of small missing studies in the lower sections of the plot, suggests that the results are unlikely to be substantially influenced by publication bias. For outcomes with fewer than 10 contributing studies (*H. pylori* eradication rate and *Bifidobacterium* and *Lactobacillus* abundance), a formal assessment of publication bias was not performed in accordance with the Cochrane Handbook recommendations.

## 4. Discussion

### 4.1. Principal Findings and Clinical Significance

To the best of our knowledge, this is the first systematic review to specifically evaluate the effects of herbal medicines and natural products on the gut microbiota of patients receiving PPI-containing therapies [16]. These findings generally suggest that herbal and natural product interventions may contribute to improved gut microbial homeostasis in this pharmacologically complex population, with concurrent favorable effects on clinical outcomes, including *H. pylori* eradication rates, gastrointestinal symptom scores, and quality of life.

Across the nine studies reporting extractable *H. pylori* eradication data, eight demonstrated the statistically significant superiority of herbal adjunct therapy over bismuth quadruple therapy alone. Specifically, one trial [32] reported 97.78% vs. 88.89%, another investigation [39] reported 95.83% vs. 79.17%, and another [44] reported 96.55% vs. 86.21%. One study [46] reported a numerically higher but non-significant difference (86.67% vs. 83.33%; *p* > 0.05), likely reflecting the limited statistical power (n = 30 per group) and short treatment duration (14 days). Meta-analysis of all nine studies demonstrated a significantly higher pooled *H. pylori* eradication rate in herbal intervention groups (pooled RR = 1.20, 95% CI 1.14–1.27; *I*^2^ = 33%), with low-to-moderate heterogeneity. This clinical advantage is particularly significant given the rising global rates of antibiotic resistance to standard quadruple therapies [21]. Recurrence data were reported in two studies: one trial [32] reported a 6-month recurrence rate of 1.15% versus 10.39% in favor of the herbal adjunct group, whereas another study [46] similarly reported a lower recurrence rate in the herbal group (16.67% vs. 47.83%), although the *H. pylori* eradication rate difference in that investigation was not statistically significant. This reduction in recurrence suggests that herbal interventions may offer a more durable therapeutic effect, potentially through the restoration of the gastric and intestinal ecological niche via herb-microbiota interactions [48]. Considering the limited number of studies reporting recurrence outcomes, these findings should be interpreted with caution and reported descriptively.

The Chinese-style clinical total effective rates were consistently higher in the herbal intervention groups across the 12 eligible studies. Meta-analysis confirmed a significantly higher pooled total effective rate in herbal intervention groups (pooled RR = 1.19, 95% CI 1.14–1.25; *I*^2^ = 0%). However, because this outcome was derived from Chinese-style composite response criteria and most contributing trials were open-label, the apparent absence of statistical heterogeneity should not be interpreted as definitive evidence of clinically uniform treatment effects. The magnitude of the individual study benefits ranged from 12 to 23%, suggesting possible additive clinical value of herbal medicines beyond standard pharmacological therapy. Benefits were also observed in GERD/NERD populations, where herbal interventions demonstrated either superiority or non-inferiority compared with PPI therapy, alongside additional improvements in quality-of-life measures, which are particularly relevant given the well-recognized limitations of PPI monotherapy in this population [3,5,49]. The Chinese-style total effective rate should be interpreted with caution. Although commonly used in Chinese clinical trials of herbal medicine, this composite endpoint is not directly comparable to internationally standardized symptom or quality-of-life measures. Moreover, because most studies contributing to this outcome were open-label, performance and outcome assessment bias may have contributed to the apparently consistent treatment effects. Accordingly, this outcome was considered supportive rather than confirmatory evidence.

### 4.2. Gut Microbiota Modulation: Diversity and Ecosystem-Level Effects

A central finding of this review is that herbal and natural product interventions may contribute to the preservation of gut microbial homeostasis under conditions of pharmacologically induced dysbiosis. Across studies employing high-resolution 16S rRNA/rDNA sequencing (n = 8 studies), these interventions were associated with maintenance or improvement of α-diversity, whereas control groups receiving PPI-based or quadruple therapy frequently demonstrated reduced diversity, a commonly used indicator of microbial richness and ecological stability [1,2]. The maintenance of α-diversity observed in herbal groups is crucial, as higher microbial richness is a known determinant of ecological resilience against exogenous perturbations, such as broad-spectrum antibiotics [50,51].

Importantly, the alterations in the microbiota observed in this review cannot be solely attributed to acid suppression. In many studies, particularly those involving *H. pylori* eradication, patients were concurrently exposed to broad-spectrum antibiotics known to induce profound microbiota disruption [9,10]. Therefore, the observed dysbiotic patterns reflect a composite effect of both acid suppression and antibiotic exposure, a distinction explicitly specified in the Section 2 of this review. Given that non-antibiotic drugs such as PPIs can exert antibiotic-like pressures on the gut microbiome [52], combined exposure to both represents a significant ecological challenge that necessitates the adjunctive protective effects of natural products. This distinction has direct clinical relevance because these interventions in eradication settings should be interpreted as adjunctive strategies within a composite pharmacological context rather than as interventions targeting PPI-induced dysbiosis alone. This issue was pre-specified in the study protocol [16] and addressed through planned subgroup analyses stratified by antibiotic exposure status; however, formal subgroup meta-analyses were limited by the small number of studies and non-overlapping outcome structures between PPI monotherapy and bismuth quadruple therapy contexts.

At the β-diversity level, one investigation [43] provided particularly informative data. Principal coordinate analysis demonstrated that the gut microbiome of patients with NERD was significantly different from that of healthy controls at baseline. After eight weeks of Hewei Jiangni recipe treatment, the microbiome configuration of the TCM group converged toward the healthy control profile, a difference that was no longer statistically significant, whereas the omeprazole group retained a microbial structure significantly divergent from healthy controls. This pattern suggests that herbal medicine may contribute to a microbiome configuration that more closely resembles that of healthy controls, rather than merely attenuating dysbiosis.

Another trial [34] further demonstrated that Poliprotect caused no significant changes in α- or β-diversity, whereas the omeprazole group showed a significant increase in Bray–Curtis dissimilarity from baseline, with specific enrichment of oral-derived genera (*Streptococcus salivarius*, *Streptococcus sinensis*, *Haemophilus parainfluenzae*, *Veillonella dispar*). This finding illustrates that even in the absence of antibiotic co-administration, PPI use alone can induce measurable microbiome perturbation, whereas the natural preparation was not associated with comparable microbiome perturbation.

The specific enrichment of orally derived taxa observed in the omeprazole group aligns with the well-established theory that acid suppression facilitates the translocation of oral microbiota to the lower gastrointestinal tract [49,53]. Nevertheless, these microbiome findings should be considered exploratory and hypothesis-generating rather than confirmatory evidence of microbiota restoration, given the substantial heterogeneity across studies in sequencing methods, disease populations, intervention composition, and outcome reporting.

### 4.3. Pathobiont Suppression and Microbial Stability

A consistent finding was the preservation or increase in beneficial taxa, particularly *Bifidobacterium* and *Lactobacillus*, in the herbal intervention groups. Notably, in several studies, control groups receiving quadruple therapy exhibited a reduction in these taxa, reflecting antibiotic-induced dysbiosis [54], whereas herbal adjunct therapy prevented this decline and promoted microbial recovery. For example, in one trial [39], *Bifidobacterium* concentrations decreased from 7.66 to 6.77 lgCFU/g in the control group, while increasing from 7.72 to 9.13 lgCFU/g in the herbal group. A parallel pattern of decline was observed in another investigation [41]. An exploratory meta-analysis of four main studies confirmed significantly higher post-treatment *Bifidobacterium* levels in herbal intervention groups (pooled SMD = 1.39), with a parallel finding for *Lactobacillus* (pooled SMD = 1.91). Considerable heterogeneity was observed for both outcomes (*I*^2^ = 96–98%), likely attributable to differences in measurement platforms and disease populations, and sensitivity analyses incorporating two additional studies yielded directionally consistent results. Although the meta-analysis yielded statistically significant results, a high degree of heterogeneity warrants cautious interpretation of the pooled estimates. Taken together, these findings suggest that the interventions may help buffer microbiota disruption and support beneficial microbial populations.

Beyond beneficial taxon enrichment, these interventions have also been associated with the suppression of potentially pathogenic or opportunistic microorganisms. Control groups frequently exhibited increased relative abundance of genera such as *Escherichia-Shigella*, *Enterococcus*, and *Staphylococcus*, which are commonly associated with dysbiosis and mucosal inflammation. Suppression of these pathogens is critical because their blooming following antibiotic exposure has been associated with sustained disruption of the microbial community structure and impaired ecological recovery [54,55]. In contrast, adjunctive herbal or natural product interventions attenuated or prevented these shifts. Particularly notable was the observation from one investigation [38] that the combined fucoidan plus synbiotics group achieved complete suppression of *Escherichia-Shigella* proliferation, a finding not replicated in the fucoidan-alone or synbiotics-alone groups, suggesting a synergistic effect of combined fucoidan and synbiotic strategies, consistent with the established prebiotic properties of fucoidan in promoting beneficial commensal enrichment [56].

These findings suggest that the microbiota-modulating effects of these interventions may extend beyond the simple enrichment of beneficial bacteria and encompass broader ecosystem-level regulation, including the maintenance of microbial balance and prevention of pathobiont overgrowth.

### 4.4. Synbiotic-like Combinations and Additive Effects

Two studies [33,38] incorporated interventions that conceptually aligned with the synbiotic paradigm [57]: simultaneous provision of prebiotic-like herbal substrates and probiotic microorganisms. One study [33] combined an herbal capsule with a *Bifidobacterium*-*Lactobacillus* preparation, whereas another trial [38] evaluated fucoidan with a commercially defined synbiotic formulation.

In the latter study [38], only the combined fucoidan and synbiotics group achieved complete suppression of *Escherichia-Shigella* proliferation and a significant reduction in *Klebsiella*, effects not observed in the fucoidan-alone or synbiotics-alone groups, suggesting a synergistic interaction. Although the small per-group sample size (n = 20) limits definitive conclusions, these findings support the potential of herbal-probiotic combination strategies. However, the interpretation of the former investigation [33] is limited by the fact that the observed microbiome-protective effect cannot be attributed solely to the herbal component, as it was co-administered with a *Bifidobacterium*-*Lactobacillus* probiotic preparation, precluding the isolation of the herbal contribution to microbiome outcomes.

### 4.5. Mechanistic Insights: Microbiome-Host Interactions

The mechanisms underlying the observed effects are likely multifactorial and reflect the complex, multicomponent nature of herbal and natural product interventions. One key mechanism is the prebiotic-like effect of herbal polysaccharides and other bioactive compounds, which serve as fermentation substrates for commensal bacteria, promoting the selective enrichment of beneficial taxa, including butyrate-producing genera (*Faecalibacterium*, *Roseburia*, *Blautia*). This supports intestinal barrier integrity and dampens mucosal inflammation through short-chain fatty acid (SCFA)-mediated pathways [14,15].

Two studies incorporated metabolomic analyses alongside microbiome sequencing. One investigation [31] identified a correction of the glutamate metabolism pathway in the herbal intervention group, an effect that was not observed with omeprazole monotherapy. Another trial [43] demonstrated that the herbal formula reversed three disease-associated metabolic pathways in patients with NERD (tryptophan metabolism, glycine/serine/threonine metabolism, and neuroactive ligand-receptor interaction), with the normalization of three key differentially expressed metabolites to healthy control levels. These findings provide mechanistic evidence that the symptomatic benefits of these interventions may be mediated through modulation of the gut–brain metabolic axis [58].

Furthermore, a bidirectional interaction exists in which the gut microbiota metabolizes herbal compounds into bioactive metabolites, which in turn regulate the microbial composition and host physiology [14,15]. This reciprocal modulation distinguishes these interventions mechanistically from both conventional acid suppression and single-strain probiotic supplementation [12,13,59].

### 4.6. Comparison with Probiotic-Based Strategies

Unlike probiotic supplementation, which typically introduces a limited number of exogenous strains with strain-specific and context-dependent effects [9,11], herbal and natural product interventions may broadly reshape the microbial ecosystem by providing diverse bioactive compounds that simultaneously act as substrates for multiple microbial taxa [59,60]. This ecosystem-level modulation may explain the consistent preservation of microbial diversity and enrichment of SCFA-producing bacteria observed across the studies in this review.

Rather than introducing exogenous microorganisms, these interventions may enhance the resilience and recovery capacity of the endogenous microbiota, promoting restoration following pharmacological perturbation. Evidence supporting this concept includes the β-diversity normalization observed in one investigation [43], the consistent enrichment of diverse butyrate-producing genera across multiple studies, and the comparative microbiome stability observed in another trial [34] with the natural preparation versus omeprazole. These differences suggest that these interventions may represent a strategy complementary to conventional probiotic approaches, particularly in contexts where broad-spectrum antibiotic co-administration renders single-strain supplementation insufficient.

### 4.7. Safety Profile

Overall, the herbal and natural product interventions demonstrated favorable safety profiles. Across studies reporting adverse events, the incidence of gastrointestinal side effects was generally lower than or comparable to that observed in control groups receiving standard pharmacological therapy.

However, one study [31] reported an important safety signal documenting liver enzyme abnormalities in three participants receiving a herbal intervention combined with omeprazole, including two with moderate-to-severe elevations. This finding warrants careful hepatic safety monitoring in future trials involving multiherbal formulations co-administered with hepatically metabolized drugs [61]. Given that PPIs and certain herbal compounds share cytochrome P450 metabolic pathways [62,63], the potential for herb-drug interactions should be rigorously evaluated to ensure patient safety. The definitions and reporting practices for adverse events vary across studies, limiting direct cross-study comparisons.

### 4.8. Methodological Considerations and Heterogeneity

Several methodological considerations must be acknowledged. The methodological qualities of the included studies varied considerably. Three studies [31,34,43] were rated as having a low overall risk of bias and employed double-blind, double-dummy designs with adequate allocation concealment. Aside from these three low-risk studies, the remaining randomized trials were judged to have some concerns, mainly because of their open-label design and limited reporting of allocation concealment. The single-arm pre-post study [30] was assessed separately using the ROBINS-I and rated as moderate-risk.

The absence of blinding, which is inherently challenging in studies involving Chinese herbal decoctions because of their distinctive taste and appearance, was the predominant source of some concerns and judgments. Therefore, performance bias cannot be excluded in open-label designs, particularly for subjectively assessed outcomes such as symptom scores.

Substantial heterogeneity was observed across the studies in terms of disease populations, intervention compositions, treatment duration (14 days to 12 weeks), and microbiome assessment methods (16S rRNA sequencing, RT-PCR/RT-qPCR, culture-based enumeration, and MALDI-TOF mass spectrometry). These differences necessitated narrative synthesis for several outcomes and limited the direct quantitative pooling of microbiome diversity indices [62].

However, the confounding effect of antibiotic exposure is a critical limitation. In studies involving *H. pylori* eradication therapy, the observed microbiota changes reflect the combined effect of acid suppression and broad-spectrum antibiotic use and cannot be attributed solely to PPI-induced dysbiosis. This distinction was explicitly pre-specified in the study protocol [16] and incorporated into planned subgroup analyses stratified by antibiotic exposure status. In addition, most pooled clinical outcomes were derived predominantly from open-label studies judged as having “some concerns” in the RoB 2.0 assessment. Therefore, the apparent consistency of some pooled effects should not be interpreted as definitive evidence of robust clinical efficacy.

### 4.9. Limitations

This study had several limitations. First, most of the included studies were conducted in China and evaluated traditional Chinese herbal formulations, which may limit the generalizability of the findings to non-Asian populations and healthcare systems in which these preparations are not routinely available. The single non-Asian study conducted in Australia was a pre-post observational design and contributed only to the narrative synthesis. Second, publication bias cannot be excluded, as studies with statistically significant positive outcomes are more likely to be published. A funnel plot was generated for the total effective rate outcome, and visual inspection suggested approximate symmetry; however, formal statistical testing was not performed. Third, the short study durations (predominantly 2–4 weeks) preclude conclusions regarding the durability of microbiome effects following treatment cessation, which is a critical consideration for conditions characterized by frequent relapses. Fourth, the absence of standardized microbiome outcome reporting across studies, including inconsistent use of α-diversity indices and varying taxonomic resolution, complicated cross-study comparisons; future trials would benefit from adherence to established reporting standards such as the STORMS checklist for microbiome studies [29]. Fifth, dietary intake patterns were not systematically reported or controlled across the included studies and may therefore have acted as important confounders of microbiome-related outcomes. Given the well-established influence of diet on gut microbiota composition, this limitation should be considered when interpreting taxonomic and diversity-related findings [64]. Sixth, the reliance on 16S rRNA sequencing in most studies limits taxonomic resolution to the genus level, precluding strain-specific insights that may be critical for mechanistic interpretation [65]. Finally, the pharmacological complexity of diverse herbal formulations makes it difficult to isolate the precise bioactive constituents driving the observed synergistic effects [48,59].

## 5. Conclusions

This systematic review provides preliminary evidence that herbal medicines and natural products may contribute to favorable gut microbiota modulation and improved clinical outcomes in patients receiving PPI-containing therapies. In most included studies, herbal adjunct therapy was associated with higher *H. pylori* eradication rates, improved clinical outcomes, attenuation of antibiotic-associated microbiota disruption, and an acceptable safety profile. However, these findings should be interpreted cautiously because many included studies involved composite pharmacological exposures, particularly bismuth quadruple therapy, and the certainty of evidence was limited by methodological concerns, outcome indirectness, and heterogeneity. Most pooled outcomes were supported primarily by studies with open-label designs and moderate methodological limitations. Importantly, these findings suggest that these interventions may act at the level of microbial ecosystem regulation, potentially offering a mechanistically distinct approach to managing pharmacologically induced dysbiosis that differs from both conventional acid suppression and single-strain probiotic supplementation. Certain interventions, particularly when combined with probiotic supplementation in a synbiotic-like strategy, have shown potential for pathobiont suppression and preservation of beneficial communities; however, these microbiome findings remain exploratory and require confirmation in larger, adequately powered trials. Future studies with stratified analyses according to antibiotic exposure status are warranted to better disentangle the independent contributions of acid suppression, antibiotic co-administration, and underlying disease context. High-quality multicenter randomized trials with standardized microbiome assessments, longer follow-up periods, and pre-specified GRADE-based outcome hierarchies are needed to consolidate the evidence base and establish the role of these interventions in integrative therapeutic guidelines. These findings may have implications for integrative therapeutic strategies targeting treatment-induced dysbiosis.

## Figures and Tables

**Table 1:** Characteristics of included studies.

Study	Country	Study Design	Disease/Condition	Intervention	Comparator	n (Int/ Con)	Duration	Microbiome Assessment (Sample)	Antibiotic Exposure
Ried et al., 2020 [30]	Australia	Pre-post single-arm	GI disorders (GERD/IBS)	NC Gut Relief Formula (curcumin, Aloe vera, slippery elm, guar gum)	No comparator (self-control)	43 (43/–)	16 wk	GI Effects PCR (fecal)	No
Zhang et al., 2021 [31]	China	RCT (multicenter, double-blind)	NERD	JianpiQinghua granule + omeprazole 10 mg/d	Omeprazole 20 mg/d	204 (102/102)	4 wk	16S rRNA seq. (fecal)	No
Li et al., 2022 [32]	China	RCT (prospective)	*H. pylori*-associated chronic gastritis	Weian Chuyou Decoction + bismuth quadruple therapy	Bismuth quadruple therapy alone	180 (90/90)	6 wk	Culture-based (fecal)	Yes
Yang et al., 2023 [33]	China	RCT	*H. pylori*-associated chronic gastritis	Jinghua Weikang Capsule + *Bifidobacterium*/*Lactobacillus* + bismuth quadruple therapy	Bismuth quadruple therapy alone	130 (65/65)	2 wk	Culture-based (fecal)	Yes
Corazziari et al., 2023 [34]	Italy	RCT (multicenter, double-blind)	Heartburn/EPS (NERD/functional dyspepsia)	Poliprotect (Aloe vera, Malva sylvestris, Althaea officinalis)	Omeprazole 20 mg/d	275 (131/126)	4 wk (+4 wk open)	16S rRNA seq. (fecal)	No
Wang et al., 2023 [35]	China	RCT (3-arm, open-label)	*H. pylori* infection	Fucoidan 1000 mg bid + bismuth quadruple therapy (simultaneous or sequential)	Bismuth quadruple therapy alone	90 (SF = 30, FS = 30/30)	14–56 d (arm-dependent)	16S rDNA seq. (fecal)	Yes
Zhang et al., 2024 [36]	China	RCT	Refractory GERD (rGERD)	Shugan Hewei Formula bid	Esomeprazole 40 mg/d + mosapride	80 (40/40)	8 wk	16S rDNA seq. (fecal)	No
Huang, 2024 [37]	China	RCT	*H. pylori*-associated chronic gastritis	Modified Sijunzi Decoction + bismuth quadruple therapy	Bismuth quadruple therapy alone	86 (43/43)	2 wk	16S rDNA seq. (fecal)	Yes
Wang et al., 2024 [38]	China	RCT (4-arm, open-label)	*H. pylori* infection	Fucoidan ± synbiotics + bismuth quadruple therapy (QF/QS/QFS)	Bismuth quadruple therapy alone (QT)	80 (each arm n = 20)	Antibiotics 2 wk; supplements 6 wk	16S rRNA seq. (fecal)	Yes
Shi et al., 2025 [39]	China	RCT	*H. pylori* infection (damp-heat type)	Lianren Huashi Granule + bismuth quadruple therapy	Bismuth quadruple therapy alone	100 (48/48)	2 wk	RT-PCR (fecal)	Yes
Zhu and Tang, 2025 [40]	China	RCT (prospective)	*H. pylori*-positive gastric ulcer	Longqi Weikang Tablet + bismuth quadruple therapy	Bismuth quadruple therapy alone	89 (45/44)	2 wk	Culture-based (automated; fecal)	Yes
Zhang et al., 2025 [41]	China	RCT	*H. pylori*-associated chronic gastritis	Qinggan Jieyou Prescription + bismuth quadruple therapy	Bismuth quadruple therapy alone	89 (44/45)	2 wk	RT-PCR (fecal)	Yes
Li et al., 2025 [42]	China	RCT (prospective)	Chronic gastritis + *H. pylori* infection	Wenzhong Hewei Decoction + bismuth quadruple therapy	Bismuth quadruple therapy alone	116 (58/58)	4 wk	Gastric microbiota % (gastric fluid)	Yes
Zhang et al., 2025 [43]	China	RCT (single-center, double-blind)	NERD (cold-heat mixed type)	Hewei Jiangni Recipe (HWJNR)	Omeprazole 20 mg/d + placebo HWJNR	72 (30/30)	8 wk	16S rRNA seq. + metabolomics (fecal)	No
Liu et al., 2026 [44]	China	RCT	Chronic gastritis + *H. pylori* infection	Yiwei Decoction + Maimendong Decoction + bismuth quadruple therapy	Bismuth quadruple therapy alone	116 (58/58)	4 wk	RT-qPCR (fecal)	Yes
Liu et al., 2026 [45]	China	RCT	Chronic atrophic gastritis + intestinal metaplasia	Shihu Xiaowei Decoction + standard quadruple therapy	Standard quadruple therapy alone	110 (55/55)	4 wk	MALDI-TOF MS (Bruker; fecal)	Yes
Jia et al., 2026 [46]	China	RCT (prospective)	Chronic non-atrophic gastritis + *H. pylori* infection	Huashi Qingyou Formula + bismuth quadruple therapy	Bismuth quadruple therapy alone	60 (30/30)	14 d	16S rRNA seq. (fecal)	Yes
Zhao and Sheng, 2026 [47]	China	RCT	*H. pylori*- positive erosive gastritis	Banxia Xiexin Decoction + quadruple therapy	Quadruple therapy alone	64 (32/32)	2 wk	RT-qPCR (fecal)	Yes

RCT = randomized controlled trial; NERD = non-erosive reflux disease; GERD = gastroesophageal reflux disease; rGERD = refractory GERD; EPS = epigastric pain syndrome; *H. pylori* = *Helicobacter pylori*; seq. = sequencing; PCR = polymerase chain reaction; RT-qPCR = real-time quantitative PCR; MALDI-TOF MS = matrix-assisted laser desorption/ionization time-of-flight mass spectrometry; wk = weeks; d = days; Int = intervention; Con = control; n = number of participants. Li et al., 2025 [42] assessed gastric microbiota (percentage composition from gastric fluid/biopsy), not fecal gut microbiota, and were therefore analyzed separately in the narrative synthesis. Wang et al., 2023 [35] was a three-arm trial, and Wang et al., 2024 [38] was a four-arm trial. For multi-arm trials, only one pairwise comparison was used for the primary analysis; additional arms were included in sensitivity analyses only. Numbers in parentheses reflect the main analysis arms.

**Table 2:** GRADE Summary of Findings for Primary and Exploratory Outcomes.

Outcomes	No. of Studies	Certainty of Evidence (GRADE)	Main Reasons for Downgrading
H. pylori eradication rate	9 RCTs	Low	Risk of bias (open-label design), indirectness
Total effective rate (Chinese-style)	12 RCTs	Very low	Indirectness (non-standardized outcome), risk of bias
*Bifidobacterium* abundance	4 RCTs	Very low	Inconsistency (high heterogeneity, *I*^2^ = 98%), risk of bias
*Lactobacillus* abundance	4 RCTs	Very low	Inconsistency (high heterogeneity, *I*^2^ = 96%), risk of bias

**Table 3:** *H. pylori* eradication rates across included studies.

Study	Int n	Int ER%	Con n	Con ER%	*p* Value	Method
Li et al., 2022 [32]	90	97.78% (88/90)	90	88.89% (80/90)	*p* = 0.017	Culture-based
Yang et al., 2023 [33]	65	80.00% (52/65)	65	53.33% (35/65)	*p* < 0.05	Culture-based
Shi et al., 2025 [39]	48	95.83% (46/48)	48	79.17% (38/48)	*p* = 0.014	RT-PCR
Zhu and Tang, 2025 [40]	45	91.11% (41/45)	44	72.73% (32/44)	*p* = 0.024	Culture
Zhang et al., 2025 [41]	44	93.2% (41/44)	45	77.8% (35/45)	*p* < 0.05	RT-PCR
Li et al., 2025 [42]	58	87.93% (51/58)	58	68.97% (40/58)	*p* = 0.013	Breath test
Liu et al., 2026 [44]	58	96.55% (56/58)	58	86.21% (50/58)	*p* = 0.047	RT-qPCR
Jia et al., 2026 [46]	30	86.67% (26/30)	30	83.33% (25/30)	*p* > 0.05 (NS)	16S rRNA
Zhao and Sheng, 2026 [47]	32	90.63% (29/32)	32	68.75% (22/32)	*p* = 0.030	RT-qPCR

Int = intervention; Con = control; ER = eradication rate; NS = not significant. Li et al., 2025 [42], measured gastric (not fecal) microbiota. Jia et al., 2026 [46], included for completeness; non-significant between-group difference.

**Table 4:** Chinese-style total effective rate.

Study	Int ER (n/N)	Con ER (n/N)	*p* Value	Disease
Li et al., 2022 [32]	96.67% (87/90)	87.78% (79/90)	*p* = 0.026	*H. pylori* gastritis
Yang et al., 2023 [33]	96.67% (63/65)	80.00% (52/65)	*p* = 0.004	*H. pylori* gastritis
Zhang et al., 2024 [36]	82.50% (33/40)	65.00% (26/40)	*p* = 0.008	rGERD
Huang, 2024 [37]	90.70% (39/43)	72.09% (31/43)	*p* = 0.027	*H. pylori* gastritis
Shi et al., 2025 [39]	93.75% (45/48)	79.17% (38/48)	*p* < 0.05	*H. pylori* infection
Zhu and Tang, 2025 [40]	95.56% (43/45)	79.55% (35/44)	*p* = 0.022	*H. pylori* gastric ulcer
Zhang et al., 2025 [41]	93.2% (41/44)	77.8% (35/45)	*p* < 0.05	*H. pylori* gastritis
Li et al., 2025 [42]	91.38% (53/58)	75.86% (44/58)	*p* = 0.024	*H. pylori* + chronic gastritis
Liu et al., 2026 [44]	94.83% (55/58)	79.31% (46/58)	*p* = 0.013	*H. pylori* + chronic gastritis
Liu et al., 2026 [45]	94.55% (52/55)	80.00% (44/55)	*p* = 0.022	CAG + intestinal met
Jia et al., 2026 [46]	100.00% (30/30)	76.67% (23/30)	*p* < 0.05	CNAG + *H. pylori*
Zhao and Sheng, 2026 [47]	96.88% (31/32)	81.25% (26/32)	*p* = 0.045	*H. pylori* erosive gastritis

All 12 studies reported statistically significant differences favoring the herbal intervention group. rGERD = refractory GERD; CAG = chronic atrophic gastritis; CNAG = chronic non-atrophic gastritis.

**Table 5:** *Bifidobacterium* abundance.

Study	n/Grp	Int (Pre → Post)	Con (Pre → Post)	Between-Group *p*	Method
Shi et al., 2025 * [39]	48	7.72 ± 0.86 → 9.13 ± 0.65	7.66 ± 0.73 → 6.77 ± 0.69 ↓	*p* < 0.001	RT-PCR
Zhang et al., 2025 * [41]	44	7.44 ± 0.34 → 8.67 ± 0.55	7.37 ± 0.45 → 6.49 ± 0.51 ↓	*p* < 0.05	RT-PCR
Liu et al., 2026 * [45]	55	7.02 ± 1.64 → 8.79 ± 1.17	7.41 ± 1.73 → 8.28 ± 1.34	*p* = 0.036	MALDI-TOF
Zhao and Sheng, 2026 * [47]	32	7.45 ± 0.52 → 9.36 ± 1.04	7.61 ± 0.55 → 8.67 ± 1.12	*p* = 0.013	RT-qPCR
Li et al., 2022 † [32]	90	5.11 ± 0.39 → 7.39 ± 0.35	5.20 ± 0.42 → 6.81 ± 0.40	*p* < 0.001	Culture
Liu et al., 2026 † [44]	58	9.05 ± 0.70 → 11.89 ± 0.84	9.02 ± 0.75 → 10.53 ± 0.79	*p* < 0.05	RT-qPCR
Zhu and Tang, 2025 ‡ [40]	45	7.10 ± 0.62 → 8.08 ± 0.96	7.05 ± 0.68 → 7.72 ± 0.81	*p* = 0.059 (NS)	Culture

* = main exploratory analysis; † = sensitivity analysis only; ‡ = excluded from pooling (non-significant between-group difference). ↓ = control group showed a decrease. Units were reported as log-transformed abundance measures (lgCFU/g or logCFU/g, depending on study and measurement platform).The symbol “→” indicates the change from pre-treatment to post-treatment. NS = not statistically significant.

**Table 6:** *Lactobacillus* abundance.

Study	n/Grp	Int (Pre→Post)	Con (Pre→Post)	Between-Group *p*	Method
Shi et al., 2025 * [39]	48	7.81 ± 0.67 → 8.56 ± 0.47	7.76 ± 0.55 → 6.83 ± 0.58 ↓	*p* < 0.001	RT-PCR
Zhang et al., 2025 * [41]	44	7.17 ± 0.58 → 8.22 ± 0.63	7.29 ± 0.61 → 6.46 ± 0.52 ↓	*p* < 0.05	RT-PCR
Liu et al., 2026 * [45]	55	6.48 ± 1.14 → 8.39 ± 1.05	6.06 ± 1.27 → 7.47 ± 1.24	*p* < 0.001	MALDI-TOF
Zhao and Sheng, 2026 * [47]	32	7.77 ± 0.54 → 9.19 ± 0.97	7.82 ± 0.64 → 8.56 ± 1.05	*p* = 0.015	RT-qPCR
Li et al., 2022 † [32]	90	5.11 ± 0.35 → 6.73 ± 0.38	5.20 ± 0.37 → 6.21 ± 0.35	*p* < 0.001	Culture
Liu et al., 2026 † [44]	58	9.19 ± 0.68 → 11.74 ± 0.81	9.13 ± 0.72 → 10.41 ± 0.89	*p* < 0.05	RT-qPCR
Zhu and Tang, 2025 ‡ [40]	45	NR → NR	NR → NR	*p* = 0.145 (NS)	Culture

* = main exploratory analysis; † = sensitivity; ‡ = excluded. ↓ = control group decrease. NR = not reported. Units were reported as log-transformed abundance measures (lgCFU/g or logCFU/g, depending on study and measurement platform). NS = not statistically significant.

## Data Availability

No new data were created or analyzed in this study. Data sharing is not applicable to this article.

## Appendix A

**Table A1:** Search strategies for each database.

Database	Search Strategy	Results (n)
PubMed/MEDLINE	(“Proton Pump Inhibitors”[MeSH] OR “proton pump inhibitor*”[All Fields] OR “PPI”[All Fields] OR “acid suppression”[All Fields] OR “omeprazole”[All Fields] OR “esomeprazole”[All Fields] OR “lansoprazole”[All Fields] OR “pantoprazole”[All Fields] OR “rabeprazole”[All Fields] OR “h pylori eradication”[All Fields] OR “quadruple therapy”[All Fields] OR “bismuth therapy”[All Fields]) AND (“Gastrointestinal Microbiome”[MeSH] OR “Microbiota”[MeSH] OR “Dysbiosis”[MeSH] OR “microbiota*”[All Fields] OR “microbiome*”[All Fields] OR “gut flora”[All Fields] OR “dysbiosis”[All Fields] OR “microflora”[All Fields]) AND (“Medicine, Chinese Traditional”[MeSH] OR “Drugs, Chinese Herbal”[MeSH] OR “Herbal Medicine”[MeSH] OR “Plants, Medicinal”[MeSH] OR “herbal”[All Fields] OR “botanical”[All Fields] OR “natural product*”[All Fields] OR “extract*”[All Fields] OR “phytotherap*”[All Fields] OR “decoction”[All Fields] OR “formula*”[All Fields] OR “polysaccharide*”[All Fields])	227
EMBASE	(‘proton pump inhibitor*’ OR PPI OR omeprazole OR esomeprazole OR lansoprazole OR pantoprazole OR rabeprazole OR ‘acid suppression’ OR ‘eradication therapy’ OR ‘quadruple therapy’) AND (‘gut microbiota’ OR microbiome OR microbiota OR microflora OR dysbiosis OR ‘gut flora’) AND (herbal OR ‘herbal medicine’ OR phytotherapy OR ‘traditional chinese medicine’ OR ‘natural product*’ OR botanical OR extract OR polysaccharide OR decoction)	184
Web of Science	TS = ((“proton pump inhibitor*” OR PPI OR omeprazole OR esomeprazole OR lansoprazole OR pantoprazole OR rabeprazole OR “acid suppression” OR “eradication therapy” OR “quadruple therapy”) AND (“gut microbiota” OR microbiome OR microbiota OR microflora OR dysbiosis) AND (herbal OR “herbal medicine” OR decoction OR formula OR phytotherapy OR “traditional Chinese medicine” OR “natural product*” OR botanical OR extract OR polysaccharide))	137
Scopus	TITLE-ABS-KEY((“proton pump inhibitor*” OR PPI OR omeprazole OR esomeprazole OR lansoprazole OR pantoprazole OR rabeprazole OR “acid suppression” OR “eradication therapy” OR “quadruple therapy”) AND (“gut microbiota” OR microbiome OR microbiota OR microflora OR dysbiosis) AND (herbal OR “herbal medicine” OR decoction OR formula OR phytotherapy OR “traditional Chinese medicine” OR “natural product*” OR botanical OR extract OR polysaccharide))	157
CNKI	(PPI OR 质子泵抑制剂 OR 抑酸 OR 四联疗法 OR 三联疗法) AND (肠道菌群 OR 肠道微生物 OR 微生物 OR 微生态 OR 菌群 OR 失调) AND (中药 OR 中医 OR 方剂 OR 汤剂 OR 天然药物 OR 植物药 OR 提取物 OR 多糖)	583
CENTRAL (Cochrane)	(proton pump inhibitor* OR PPI OR omeprazole OR esomeprazole OR lansoprazole OR pantoprazole OR rabeprazole OR “acid suppression” OR “eradication therapy” OR “quadruple therapy” OR bismuth) AND (microbiota OR microbiome OR microflora OR dysbiosis OR “gut microbiota”) AND (herbal OR “herbal medicine” OR herb OR decoction OR formula OR phytotherapy OR “traditional Chinese medicine” OR TCM OR “natural product*” OR botanical OR polysaccharide OR extract OR plant-derived)	43
Total records from databases		1331
Additional records (manual search and citation tracking)		2
Total records identified		1333

Abbreviations: CENTRAL, Cochrane Central Register of Controlled Trials; CNKI, China National Knowledge Infrastructure; MeSH, Medical Subject Headings; PPI, proton pump inhibitor; TCM, traditional Chinese medicine; TS, Topic Search (Web of Science). Search conducted from inception to March 2026. Following deduplication, 1041 unique records were screened, The asterisk (*) denotes a wildcard operator used in database searches. Chinese search terms used in CNKI were translated as follows: 质子泵抑制剂 = proton pump inhibitor; 抑酸 = acid suppression; 四联疗法 = quadruple therapy; 三联疗法 = triple therapy; 肠道菌群/肠道微生物 = gut microbiota/gut microorganisms; 微生物 = microorganisms; 微生态 = microecology; 菌群 = microbiota/flora; 失调 = dysbiosis; 中药 = Chinese herbal medicine; 中医 = traditional Chinese medicine; 方剂 = herbal formula; 汤剂 = decoction; 天然药물 = natural medicine; 植物药 = botanical medicine; 提取物 = extract; 多糖 = polysaccharide.
